# Effects of neuromuscular electrical stimulation on muscle mass and strength in critically ill patients after cardiothoracic surgery (catastim 2)

**DOI:** 10.1186/2197-425X-3-S1-A818

**Published:** 2015-10-01

**Authors:** A Fischer, A Winkler, M Spiegl, A Salamon, K Altmann, M Themessl-Huber, M Mouhieddine, A Schiferer, E-M Strasser, T Paternostro-Sluga, M Hiesmayr

**Affiliations:** Medical University of Vienna, Vienna, Austria; Kaiser Franz Josef Hospital, Vienna, Austria; Donauspital, Vienna, Austria

## Introduction

Intensive care unit acquired weakness (ICUAW) affects 24-77% of patients with an ICU stay longer than one week. Neuromuscular electrical stimulation (NMES) is a feasible therapy for neuromuscular activation in sedated patients. The effect of NMES on muscle mass and strength is unclear: Randomized controlled trials (RCT) either showed no effect or beneficial effects [1]. To date, no RCT assessed the effects of NMES in a homogenous cardiothoracic surgery patient population.

## Objectives

The objective was to investigate whether early NMES would be effective in preventing loss of muscle mass and strength in critically ill patients after cardiothoracic surgery.

## Methods

The prospective RCT Catastim 2 included 54 patients (27 in the NMES group and 27 in the control group). In the intervention group, the anterior muscles of both thighs were electrically stimulated from the first postoperative day until ICU discharge for a maximum of 14 days. In the control group, the electrodes were applied, connected to the stimulator, but no electricity was delivered. Measurement of muscle layer thickness (MLT) of the anterior muscles of the thigh using two-dimensional B-mode ultrasound was assessed every other day from postoperative day 1 to ICU discharge and at hospital discharge. Muscle strength was evaluated daily in all joints of the upper and lower extremities using the Medical Research Council (MRC) scale from postoperative day 1 to ICU discharge and at hospital discharge. The effect of NMES on MLT and MRC were each analyzed in a linear mixed model.

## Results

Mean MLT decreased by 0.07 cm [95% CI, -0.08 to - 0.05 cm] per day (*P* < .001). NMES had no significant effect on MLT (*P* > .05).

Mean MRC score depended on the day. Moreover there was a significant interaction between NMES and day (*P* < .001): The more advanced the day was, the higher the mean MRC score in the NMES group was (Table 1)

**Table 1:** Mean MRC score (271 observations).

	estimate [95% confidence interval]	P value
intercept	4.07 [3.79 to 4.36]	<.001
day	0.03 [0.005 to 0.06]	.02
NMES group	-0.39 [-0.80 to 0.009]	0.06
Control group	reference	.
day * NMES group	0.07 [0.03 to 0.11]	< .001
day * Control group	reference	.

## Conclusions

In this RCT, NMES had no overall effect but a progressively increasing effect with the duration of NMES. This indicates that NMES helps to regain muscle strength only when applied for a sufficient number of days.
